# The College of Nursing

**Published:** 1919-06-28

**Authors:** 


### THE COLLEGE OF NURSING

### (LIMITED BY GUARANTEE).

### Fourth Annual Meeting.

The annual meetings of the College of Nursing opened in Manchester on Wednesday, June 18, at the Chemistry Theatre of the University. There was an attendance of about three hundred. The Hon. Sir Arthur Stanley, G.B.E., C.B., presided, and among those present were Sir Cooper Perry, M.D., F.R.C.P. (hon. secretary); Mr. Comyns Berkeley, M.C., M.D., F.R.C.P. (hon. treasurer); Dame Sidney Browne, G.B.E., R.R.C.; Miss M. Chisholm, Q.V.J.I.N. (Dublin); Miss E. M. Cummins (Liverpool); Miss R. Cox-Davies, R.R.C. (London); Miss A. W. Gill, R.R.C. (Edinburgh); Miss Graham (Edinburgh); Miss A. Hughes; Miss E. M. Musson, R.R.C. (Birmingham); Miss M. E. Sparshott, C.B.E., R.R.C. (Manchester); Miss A. Lloyd Still, C.B.E., R.R.C. (London); Miss Mary S. Rundle, R.R.C. (secretary); Miss Gertrude Cowlin (organising secretary); and the secretaries of the Irish and Scottish Boards.

**Sir Arthur Stanley** said the first business of the meeting was to offer hearty congratulations to Miss Sparshott, who had received the C.B.E. They knew how thoroughly she deserved it, and felt especially glad that it had come at this time when they were the recipients of such splendid hospitality as that which Miss Sparshott and those who were working with her offered them that day. Sir Arthur then proceeded to make the speech reported in our issue of last week (pp. 297/8). At the conclusion he invited those present to ask questions or make remarks, which elicited from one member the statement that she had been able, through the influence of the College, to secure a rise of salary for herself and another nurse; and from Miss Cann (Matron, Norfolk and Norwich Hospital) the question whether it would not be possible to adopt the title of "Sister" for trained nurses, instead of the name "Nurse." This point, Sir Arthur suggested, might be discussed first by the local centres of the College.

**Miss Seymour Yapp** (Ashton-under-Lyne), in thanking the College on behalf of all the nurses in her district for its work for the nurses, said it had resulted in better salaries, better conditions, and shorter hours for nurses in her district, at any rate, and they were all very grateful.

The appointment of the auditors, Messrs. Barton & Co., was then proposed and seconded by Miss Cummins (Liverpool) and Miss Binns (Hull), respectively.

### THE NEWLY-ELECTED COUNCIL MEMBERS.

**The Chairman** announced the result of the election :

English and Welsh Section.

Dame Sidney Browne, G.B.E., R.R.C., Matron-in-Chief, T.F.N.S.

Sir Cooper Perry, M.A., M.D., F.R.C.P., Superintendent, Guy's Hospital.

Miss Ellen M. Musson, Matron, General Hospital, Birmingham.

Miss Geraldine Bremner, Private Nurse.

Mr. Donald MacMaster, K.C., M.P., Chairman, Standing Committee E, Nurses' Registration Bill.

Miss Margaret Cummins, Matron, Royal Infirmary, Liverpool.

Mrs. Maynard Carter, late District Nurse, Staff Nurse, and Sister, T.F.N.S. (now retired).

Miss Annie Peterkin, General Superintendent, Q.V.J.I.N.

Scottish Section.

Dr. Donald Mackintosh, C.B., M.V.O., M.B., Medical Superintendent, Western Infirmary, Glasgow.

Miss Elizabeth Edmondson, R.R.C., Matron, Royal Infirmary, Aberdeen.

Irish Section.

Sir Andrew Horne, M.D., F.R.C.P.I., Master of National Maternity Hospital, Dublin.

Miss Mary Chisholm, Inspector, Irish Branch, Q.V.J.I.N.

Continuing, he said : Thank goodness, we have got Sir Cooper Perry again. I think you will agree with me that they are a very good lot indeed. By the time I make this corresponding announcement next year the whole Council will have been elected by the nurses on the register themselves.

Aberdeen's Tribute to Manchester Centre.

**Miss Edmondson** (Royal Infirmary, Aberdeen), in seconding a vote of thanks to Sir Henry Miers for the use of the room, said it was a very happy idea to have the meeting in the North of England. They had heard much about the Manchester Centre, and they could now try to arrange their Centres in the same magnificent way as Miss Sparshott had done. She added her heartiest congratulations on the occasion of the honour which had been conferred on Miss Sparshott, than whom no one in the nursing profession was more worthy to receive it. She also expressed indebtedness to Sir Arthur Stanley for his excellent work. It seemed to her he was the captain of a great ship, a great Dreadnought, and they dreaded nothing so long as they had him.

From John O'Groats to Land's End.

**Miss Sparshott,** in returning thanks, said she only wished that the ribbon and decoration of the C.B.E. were big enough to divide up amongst all the people who had helped her to gain it. She was glad to see such a tremendous gathering there—members right from the north of Scotland, from Truro, from Brighton, and all over England, Scotland, and Ireland, so that it was a very good thing that such a central spot as Manchester had been chosen for the meeting.

**Sir Cooper Perry** (Vice-Chancellor of the University of London and Honorary Secretary to the College of Nursing), who arrived late and was asked to speak, said he had nothing to say. The Bill before Parliament was a very troublesome question. Some progress had been made, and Sir Arthur had expressed his personal views as to the best solution. Sir Cooper did not think Sir Arthur was prophesying, because he knew, but it would not surprise him if the Chairman were a true prophet.

**The Chairman :** We should not end this very satisfactory meeting in a proper way unless we expressed our thanks to Miss Sparshott. I have very much pleasure in calling on Miss Lloyd Still. A vote of thanks to the Manchester Centre and Miss Sparshott for their hospitality was then proposed and seconded by Miss Lloyd Still and Miss E. M. Musson respectively, and in reply Miss Sparshott said they were more than proud to welcome the meeting, and hoped Manchester would not live up to the reputation it seemed to have of being a very wet place. She hoped the nice weather would continue, and that the members would enjoy all the things that had been arranged for them.

A Chair of Nursing.

**Miss R. Cox-Davies** said it was hoped in connection with the College studentships to be able in the course of next year to get a great development, and to establish a Chair of Nursing at one of the women's colleges, with their own expert professor established there in the Chair of Nursing. It would mean hard work, and a great deal of help from the members of the College, but if they could once do that they would have established themselves as an educational body in a way in which nothing else could establish them.

Tea was afterwards served to the visitors as guests of the Local Centre at the Royal Infirmary. In the evening the members were the guests at a reception by the Lord and Lady Mayoress of Manchester (Alderman Wm. Kay and Mrs. Kay) at the Town Hall.

### The Manchester Conference.

The Conference was held on Thursday afternoon in the University of Manchester. It was largely attended by delegates from all parts of the country, and by nurses engaged in local institutions.

In opening the proceedings, Sir Henry Miers, Vice-Chancellor of Manchester University, extended a welcome to the Conference, and expressed the great pleasure it gave the University to act as host to such a gathering. That they were meeting in such a building, and that on the platform were two Vice-Chancellors of universities, was surely a sign of the great interest which universities were going to take in the work of the College of Nursing. Through the agency of the war the world had been brought to an appreciation of the services of nurses. Their work was no longer amateur and unorganised, and he felt he was welcoming the nucleus of a tremendously important and growing profession.

### Educational Value of Local Centres.

**Miss Brown,** R.R.C., matron of the Royal Victoria Infirmary, Newcastle-on-Tyne, read her paper upon "Local Centres and how their Usefulness may be Developed." After briefly outlining the history of the formation of the Centres, and touching upon the inauguration in most of the centres of post-graduate lectures for trained nurses, Miss Brown said that though this record was no mean one, much more remained to be done if they were to justify their existence as educational centres and to help in the reconstruction ahead.

The College of Nursing proposed to establish a uniform curriculum of training and a one-portal examination for nurses. They were all eagerly awaiting this curriculum, knowing as they did that, coming from the tried and trusted leaders of the nursing profession, working together for the common good, it was sure to be the very best that had yet been evolved; but as the College could not be expected to do all the necessary work, it appeared to her that the local centres should step in and endeavour to aid hospital authorities and others interested in nursing work to arrange for this curriculum to be carried out in a thoroughly satisfactory manner in the training-schools in their own areas. A broad-minded and generous policy was required, and the barriers that had stood between hospitals, to the detriment of common progress, should be broken down once for all. (Applause.)

The day was passed when each hospital could be a law unto itself with regard to the training of probationers, and in view of the fact that the whole hospital system was likely to undergo radical changes with the coming of the new Ministry of Health, it seemed to her that the members of each local centre should endeavour as a body to ensure, as far as they were able to do so, that the interests of the nurses in their own area, their training and general welfare, received due and careful consideration. She did not anticipate any opposition on the part of Hospital Committees either to granting facilities for better education for probationers or to giving them higher pay and shorter hours of work, as they had already, in most hospitals, shown their willingness in both directions, but a Hospital Committee, like the rest of them, could only cut its coat according to its cloth, and therefore they must take care not only that their schemes were sound, but also that they were as economical as they could possibly be without impairment of efficiency. They must also remember that Hospital Committees (at any rate those known to her) had no members with a practical working knowledge of nursing, and when they claimed as local centres of the College of Nursing to have that knowledge, the members of the Hospital Committees, as business men, would expect them not only to know exactly what teaching they did want, but also to submit for their consideration and, it was hoped, their acceptance, a good, practical scheme for carrying it out suited to the needs of each particular area.

### Problem of the Small Hospital.

There was little difficulty in providing a first-class training for nurses in the great hospitals in London and the Provinces, but there was often great difficulty with regard to the smaller training schools, which, though they might or might not give a good practical training, often could not, mainly on account of expense and lack of experienced lecturers and teachers, give a good theoretical training. These small general hospitals were undoubtedly needed in some of the smaller towns, especially in industrial districts, and as they must have probationers it seemed only right to make every effort to enable them to train them in such a way that they might become first-class nurses, and also graduates of the College of Nursing, more especially as the larger hospitals could not possibly train as many probationers as were required to fill the ranks of the nursing profession. It would be unreasonable to expect a hospital of a hundred beds or so to provide a preliminary training school, a sister tutor, qualified paid lecturers and examiners—all of whom would be required if a high standard of training was to be insisted on—for the very small number of probationers admitted. Also, if they were able to provide them, it would appear to be a great waste of time, effort, and money, which might be avoided in a great measure by organisation and co-operation. The most satisfactory and economical way of attaining the best results appeared to lie in the now accepted idea of the affiliation of small hospitals with the larger ones in the same district for the purpose of training probationers, and she would suggest co-operation somewhat on the following lines:

### The Preliminary Training School's Place in Affiliation.

1. By the establishment of a Preliminary Training School for probationers by each of the greater provincial hospitals.
2. By attaching to this school enough trained lecturers and teachers (preferably nurses specially trained for the work) to lecture and teach in the different affiliated hospitals in the group.
3. By the exchange or passing on of probationers during some period of their training from the smaller to the larger hospitals, and *vice versa.*

With regard to the first suggestion, she believed the majority of hospital committees and matrons were agreed that Preliminary Training Schools were most useful and desirable in the interests of both patients and nurses.

Some of the advantages that result from them were:

1. The patient escaped the well-meant, but often clumsy, attentions of the raw probationer.
2. The probationer did not have to attend so many lectures and classes whilst she was working hard in the wards.
3. The ward sister was saved from some of the work and anxiety inseparable from the advent of a new probationer.
4. The hospital gained in increased efficiency.
5. Unintelligent pupils and those falling distinctly below the average were weeded out and did not enter the hospital at all.

She would, therefore, advocate the establishment of a Preliminary Training School by each of the great pro-

**The College of Nursing**—(*continued*).

vincial hospitals, to which not only their own probationers, but also those from the small affiliated training schools might be sent, each hospital choosing its own probationers as at present and paying for the pupils it sent to the school, the pupils themselves paying a fee as they did in the existing Preliminary Training Schools. Possibly someone might be willing to found and endow such schools, and in view of the importance to the health and well-being of the nation of a well-trained nursing service it was difficult to imagine a better use to which the rich and generous could put their money. Personally, she saw no reason why the education authorities or the Ministry of Health should not help in such a scheme.

The schools would probably be managed by the great hospitals with which the smaller ones were affiliated, and if the committee of the hospital would allow the executive committee of the local centre to appoint one or two of their members to serve on the sub-committee managing the school, in an advisory capacity, she thought much good might result. Granted the necessary funds, it would be quite possible for the local centres to run these schools themselves. Candidates for training as nurses should be required to pass a simple entrance examination before being accepted for the Preliminary Training School, should reside in the school, and the training should last from two to three months, two months usually being considered quite long enough.

The subjects taught should be somewhat as follows: Elementary anatomy; elementary physiology; hygiene; practical nursing, including bedmaking, management of helpless patients, preparation of dressings, bandaging, splint padding, uses and care of appliances, first-aid work, etc.; practical ward work (domestic) hospital routine, prevention of waste, etc.; invalid cookery, chemistry of food, economy; and mending, marking, and care of linen. The pupils, having gone through this course of instruction, would be required to pass an examination as a test of their efficiency before entering their respective hospitals. The training school itself should be provided with a bed-room or cubicle for each pupil, also a common sitting-room and a dining-room.

The workrooms would consist of a lecture-theatre, a kitchen for cookery lessons, and classrooms, one of which should be fitted as a complete hospital ward and should contain a model of a patient such as was used in St. Thomas's Hospital. This model (an American one she believed) was so excellently constructed that it was possible by its aid for a probationer to practise almost everything that she had to do for a patient on it, including such duties as bathing, giving nasal and æsophageal feeds, washing out of bladder, syringing of ears, and giving of enemas, douches, etc. There should also be accommodation for the teaching staff and for domestics.

This plan would ensure, at any rate, a uniform preliminary curriculum. The school might have a sufficient staff of nurse lecturers specially trained as teachers, and, knowing the needs of nurses, not only to give instruction in the school, but also to go out to lecture and teach in different affiliated hospitals on different days of the week, so that an identical course of instruction might be going on in all the hospitals at the same time. They would thus also keep in touch with the nurses who had passed through the school. Each large hospital might require, in addition, a sister tutor, and several small hospitals might co-operate and have one amongst them.

### Exchange of Probationers.

She would ask for consideration of the possibility of some system of exchange of probationers between large and small hospitals during some period of their training. For instance, during their third or fourth year of training (preferably the fourth), nurses from small training-schools might be sent for six months or so into a larger one to gain wider experience and an insight into the working and routine of a big hospital, whilst the nurses from the larger hospitals would be sent to take their places in the smaller ones, and would learn something of their work and different needs—a very valuable experience for an ambitious nurse, as she would probably become matron of a small hospital first if she aspired to become matron of a big one later on.

The local centre might also aim at securing a representative (one of themselves) on all Committees dealing with the nursing side of public-health work, on all of which much useful help might be given by an experienced, capable, and broad-minded nurse. One never heard of one of these committees without at least one representative of the medical profession, but she thought it would be an exception to find one boasting a representative of the nursing profession.

They all knew that there were certain people who hold the opinion that it was the duty of hospital authorities to treat their probationers almost entirely as students, and to give them, during the three or four years' training they had signed for, instruction in all the special branches of nursing. This seemed to her a somewhat unreasonable view to take, and she considered that if a hospital gave a probationer a thoroughly good general training, and, if possible—and if she showed aptitude for it—some training in one special subject during her fourth year, it was all that could reasonably be expected, provided that the probationer was neither overworked nor underpaid, and lived under good conditions. It would also appear that if the nurses' hours were to be reduced to forty-eight or fifty a week the whole of the four years will be needed to give them a thorough general training.

### Does the Probationer Expect Too Much?

Probationers were students certainly, but they were students on different terms from those obtaining in any other profession. They had been overworked, underpaid, and sometimes insufficiently taught in the past, but it seemed to her that there is now a tendency to go too far in the opposite direction, and to spend more consideration and more money on the nurses than on the patients for whom the hospitals existed. The probationer entering a hospital received board, lodging, and uniform, was nursed when sick, and had a salary of £17 to £20 a year, so that what she received during her first year was about the equivalent of £2 a week. In addition to this, she was taught her profession. Could she expect much more whilst learning the rudiments of her work? Miss Brown thought not, but she did think that the local centres might help the probationer a great deal by arranging lectures for nurses in their fourth year of training. These nurses—who would get an eight-hour day very shortly—might be glad to add to their knowledge by attending, in their off-duty time, lectures on such subjects as institutional housekeeping, laundry work, catering, management and choosing of linen, engaging and supervision of servants, or any other subjects likely to help them in their future career. These lecturers would probably be appreciated by some of the members of the local centre also, and she certainly would not limit them to purely nursing subjects.

### Centres as Information Bureaux.

Before they finish their hospital training, nurses usually knew little or nothing of the various other branches of work open to them, and here, again, the local centre might step in and arrange for district nursing superintendents to come and tell them something of district nurses' work, its advantages and possibilities. A health visitor might give them much useful help and advice, and the same applied to infant-welfare work, nursery schools, school nursing, and other branches of work open to members of the profession. The centres might also undertake to obtain particulars regarding the whole of the work open to nurses in their area, so that information and advice might be given to those who wished for it. No centre should be considered complete until it possessed a good library of nursing books. A local centre magazine would, she thought, be much appreciated in the larger centres. It would encourage members to think and to express their opinions, and would assist in that great aim they all had in view—the union of the nursing profession. (Applause.)

**The Debate on the Local Centres and other papers is unavoidably deferred until our next issue of "The Hospital."—Ed. T.H.**

(*To be continued.*)

